# The Contribution of Histone Crotonylation to Tissue Health and Disease: Focus on Kidney Health

**DOI:** 10.3389/fphar.2020.00393

**Published:** 2020-04-03

**Authors:** Julio M. Martinez-Moreno, Miguel Fontecha-Barriuso, Diego Martín-Sánchez, Maria D. Sánchez-Niño, Marta Ruiz-Ortega, Ana B. Sanz, Alberto Ortiz

**Affiliations:** ^1^ Research Institute-Fundacion Jimenez Diaz, Autonomous University of Madrid (UAM), Madrid, Spain; ^2^ Red de Investigación Renal (REDinREN), Madrid, Spain; ^3^ School of Medicine, Autonomous University of Madrid (UAM), Madrid, Spain; ^4^ IRSIN, Madrid, Spain

**Keywords:** acute kidney injury, chronic kidney disease, epigenetics, crotonylation, histone posttranslational modification, histone deacetylase, TWEAK, nephrotoxicity

## Abstract

Acute kidney injury (AKI) and chronic kidney disease (CKD) are the most severe consequences of kidney injury. They are interconnected syndromes as CKD predisposes to AKI and AKI may accelerate CKD progression. Despite their growing impact on the global burden of disease, there is no satisfactory treatment for AKI and current therapeutic approaches to CKD remain suboptimal. Recent research has focused on the therapeutic target potential of epigenetic regulation of gene expression, including non-coding RNAs and the covalent modifications of histones and DNA. Indeed, several drugs targeting histone modifications are in clinical use or undergoing clinical trials. Acyl-lysine histone modifications (e.g. methylation, acetylation, and crotonylation) have modulated experimental kidney injury. Most recently, increased histone lysine crotonylation (Kcr) was observed during experimental AKI and could be reproduced in cultured tubular cells exposed to inflammatory stress triggered by the cytokine TWEAK. The degree of kidney histone crotonylation was modulated by crotonate availability and crotonate supplementation protected from nephrotoxic AKI. We now review the functional relevance of histone crotonylation in kidney disease and other pathophysiological contexts, as well as the implications for the development of novel therapeutic approaches. These studies provide insights into the overall role of histone crotonylation in health and disease.

## Introduction: AKI and CKD

Acute kidney injury (AKI) and chronic kidney disease (CKD) are the most severe consequences of kidney injury. AKI is commonly defined by a sudden (within 48 h) increase in serum creatinine and is usually transient (Kidney Disease, 2012). However, it may be as severe as to require renal replacement therapy and is associated with an increased risk of death. CKD is defined by abnormalities of kidney structure or function that persist for longer than three months and have consequences for health (Kidney Disease, 2013; Perez-Gomez et al., 2019). CKD may progress to end-stage kidney disease requiring renal replacement therapy and is associated with an increased risk of premature death. Indeed, CKD was among the fastest growing causes of death worldwide and it is estimated that it will become the second most common cause of death within the next century in some countries (Ortiz et al., 2019). AKI and CKD are considered interconnected syndromes, as CKD predisposes to AKI and AKI may accelerate CKD progression (Chawla et al., 2014). AKI was thought to be followed by complete recovery of kidney structure or function. However, the repair process may be unsuccessful leading to engagement of pathways promoting transition to CKD. In this regard AKI and CKD share a number of pathogenic processes, including inflammation and parenchymal cell death. Additionally, kidney injury during AKI may initiate or aggravate the processes of capillary rarefaction and fibrosis that characterize CKD (Kida et al., 2013; Polichnowski, 2018; Yang et al., 2018). The clinical care of patients with AKI or CKD is marred by a paucity of effective therapeutic approaches. Thus, there is no therapy that prevents or accelerates recovery from AKI. Additionally, only a handful of drugs have been approved to slow the progression of CKD, including renin-angiotensin system blockers for proteinuric CKD and tolvaptan for polycystic kidney disease (Perez-Gomez et al., 2015). However, they slow but do not completely prevent CKD progression and do not promote regression. Thus, novel therapeutic approaches should be developed based on an improved understanding of the cellular and molecular pathogenesis of AKI and CKD and of the drivers of the AKI-to-CKD transition.

## Epigenetic Regulation of Gene Expression

Epigenetic regulation refers to mechanisms that control gene expression without altering the nucleotide sequence. In general, epigenetic modifications are heritable during cell division although they are reversible and could be altered by age, disease, and environment. Epigenetic regulators include non-coding RNAs, and covalent modifications of histones and DNA. Histone modifications involve proteins responsible for the addition (writer), recognition (reader), and removal (eraser) of modifiers such as acyl-lysine modifications.

The main modification on DNA is methylation catalyzed by DNA methyltransferases (DNMTs). These are enzymes that methylate the 5-cytosine of CpG dinucleotides, frequently located in the first exons or near promoters (Mohtat and Susztak, 2010). DNA promoter methylation is a silencing mechanism that inhibits transcription factor binding through chromatin packaging or repressor recruitment (Beckerman et al., 2014).

Histones are positively charged and they associate with negatively charged DNA to package in condensed chromatin and form nucleosomes, which are the basic units of chromatin. Nucleosomes pack 147 DNA base pairs around a histone octamer composed of two H3-H4 histone dimers stabilized as a tetramer which is flanked by two separate H2A-H2B dimers (Luger et al., 1997; Davey et al., 2002). Histone 1 (H1) acts as a linker and completes the chromatosome by protecting internucleosomal linker DNA (Campos and Reinberg, 2009). There are more than 100 types of histone modifications which regulate gene expression, such as methylation, acetylation, phosphorylation, crotonylation, sumoylation, and ubiquitination (Susztak, 2014). However, the biological function of most of these modifications remains unknown. The best characterized consist of addition of acyl groups to specific amino acids, resulting in methylation, acetylation and crotonylation of lysine residues on histone H3.

Histone methylation requires the transfer of methyl groups to histone lysine or arginine residues by histone methyltransferases. Lysine residues can accept up to three methyl groups, resulting in mono-, di-, or trimethylated lysine, and arginine can accept up to two methyl groups, resulting in mono- or dimethylated arginine. Methyl groups can be removed by histone demethylases (Morera et al., 2016). The addition of methyl groups does not change histone charge, rather provides platforms for binding of transcription factors that may stimulate or suppress gene expression (Nguyen and Zhang, 2011).

Histone acetylation, the transfer of an acetyl group to lysine residues, changes the charge of histones and favors chromatin relaxation, facilitating binding of transcription factors to promoters and gene expression (Ramakrishnan and Pili, 2013). Histone acetylation is catalyzed by histone acetyltranferases (HAT) and it is reversible when histone deacetylases (HDAC) remove acetyl groups.

Histone lysine crotonylation (Kcr) was more recently described and consists in the transfer of crotonyl groups to lysine residues of histones, also conferring them negative charge (Tan et al., 2011). Recently, a role of histone Kcr was described in AKI (Ruiz-Andres et al., 2016a). We now review the functional relevance of histone crotonylation in tissue physiopathology with emphasis in kidney disease and the implications for the development of novel therapeutic approaches.

## Epigenetic Modifications in Renal Injury

Both AKI and CKD are associated with cell death, inflammation, and diverse degrees of fibrosis. All of these could be regulated by epigenetic modifications. DNA methylation and histone acetylation and methylation are the most studied epigenetic mechanisms in renal injury, and evidence supports their functional relevance for kidney disease (**Table 1**).

**Table 1:** Examples of changes in the overall pattern of DNA methylation or histone modifications during kidney disease.

Epigenetic modification	Overall levels in kidney	Reference
**DNA methylation (-CH3):**	**Overall increase in:** peripheral blood leukocytes from inflamed CKD G5 patients kidney in RASAL1 genes in mouse model of folic acid-induced fibrosis kidney in Klotho gene in mouse model of adenine-induced CKD	(Hunag et al., 2012; Ko et al., 2013; Zhao et al., 2017)
	**Overall decrease in**: kidney in experimental AKI induced by IRI kidney in tubules of CKD patients	(Stenvinkel et al., 2017; Bechtel et al., 2010; Zhang et al., 2017)
**Histone methylation (Km, Rm, -CH3):**	**Overall increase in:** kidney in experimental renal fibrosis induced by UUO kidney in diabetic nephropathy in uninephrectomy db/db mice	(Sayyed et al., 2010; Zhou et al., 2016; Hewitson et al., 2017)
	**Overall decrease in:** kidney in db/db mice kidney in uninephrectomy C57BL/6 mice	(Sayyed et al., 2010)
**Histone acetylation (Kac, -CO-CH3):**	**Overall increase in:** kidney in experimental AKI-CKD transition induced by IRI kidney in experimental renal fibrosis induced by UUO kidney in diabetic nephropathy in uninephrectomy db/db mice	(Sayyed et al., 2010; Zager et al., 2011; Hewitson et al., 2017)
	**Overall decrease in:** kidney: early decrease in severe IRI AKI, and recovery during reperfusion kidney in db/db mice	(Marumo et al., 2008; Sayyed et al., 2010)
**Histone crotonylation (Kcr, -CO-CH=CH-CH3)**	**Overall increase in:** kidney in experimental nephrotoxic AKI by folic acid **Changes in specific histones** peripheral blood mononuclear cells from IgA nephropathy patients: increased (HIST1H2AC, HIST1H4A, and decreased HIST1H1B)	(Ruiz-Andres et al., 2016; Lin et al., 2020)

In parenthesis, modified amino acid (K, lysine; R, arginine) and the residue added.

AKI, acute kidney injury; CKD, chronic kidney disease; Kcr, crotonylation; IRI, ischemia-reperfusion injury; UUO, unilateral ureteral obstruction.

Global kidney DNA methylation was reduced in experimental AKI induced by ischemia-reperfusion injury (IRI) or cisplatin, and this was associated to the expression of inflammatory genes (Guo et al., 2017; Zhao et al., 2017). In this regard, DNA methylation contributes to silencing the expression of the nephroprotective genes Klotho (which has anti-inflammatory and anti-fibrotic properties) and RASAL1 (which has anti-fibrotic properties) in AKI. DNMTs targeting by genetic ablation or pharmacological inhibition decreased fibrosis and prevented the AKI-to-CKD transition in part because of preserved expression of Klotho and RASAL1 (Bechtel et al., 2010; Sun et al., 2012; Tampe et al., 2017).

Histone methylation, depending on context, lysine residue or extent of methylation may activate or repress gene transcription. Overall kidney histone methylation was increased in preclinical CKD induced by unilateral ureteral obstruction (UUO) or diabetic nephropathy [reviewed in (Fontecha-Barriuso et al., 2018)]. In this regard, the expression of the histone methyl-transferase, EZH2, is increased in UUO and in CKD patients and its pharmacological inhibition reduced preclinical fibrosis (Zhou et al., 2016). Similar results were found for the histone methyl-transferase, SMYD2 in clinical and preclinical PKD (Li et al., 2017). In AKI induced by IRI or endotoxin, increased histone methylation has been linked to the increased expression of pro-inflammatory and pro-fibrotic genes (Naito et al., 2008; Zager and Johnson, 2009). Moreover, histone methylation in the MCP1 and NGAL genes was detected in urine from AKI patients (Munshi et al., 2011).

Histone acetylation generally favors gene expression and it has been extensively studied in renal injury. Global changes in kidney histone acetylation were observed in experimental AKI (e.g. IRI, endotoxemia) and CKD (UUO, diabetic nephropathy). Histone acetylation increased during renal injury in most animal models analyzed (endotoxemia, UUO, diabetic nephropathy in db/db mice) (Sayyed et al., 2010; Huang et al., 2015; Hewitson et al., 2017). During ischemia reperfusion injury (IRI), histone acetylation presents a more complex regulation, it is transiently decreased during ischemia, but it recovers, or even increases, after reperfusion, and may persist increased for 3 weeks (Marumo et al., 2008; Zager et al., 2011). The impact of histone acetylation over the expression of specific relevant genes in AKI has been also studied, including the nephroprotective genes PGC1α and Klotho, 3-hydroxy-3-methyl-glutaryl-CoA reductase (HMGCR) and the inflammatory cytokine Interleukine-6 (IL-6), among others (Naito et al., 2009; Li et al., 2010; Ruiz-Andres et al., 2016b). The functional contribution of histone acetylation to kidney injury is supported by numerous preclinical studies in AKI (e.g. cisplatin, IRI) and CKD [e.g. UUO, polycystic kidney disease (PKD), diabetic nephropathy] models, and HDAC inhibitors were frequently nephroprotective (Fontecha-Barriuso et al., 2018). Related with histone acetylation, another emergent line of research is the study of the bromodomain and extraterminal (BET) protein family. BET proteins are readers that bind to acetylated lysines of histones and facilitate binding of transcription factors. BET targeting has been beneficial in preclinical kidney disease and ongoing clinical trials of the BET inhibitor apabetalone explore kidney function as a secondary outcome (Suarez-Alvarez et al., 2017; Fontecha-Barriuso et al., 2018).

These data show the complex regulation of epigenetic modifications in kidney diseases. The overall pattern of the different DNA and histone marks suggests that epigenetic modifications could favor the differential gene expression observed in kidney disease, opening the door for therapeutic strategies targeting epigenetic modulation.

## Histone Crotonylation

The application of high-sensitivity mass spectrometry techniques recently identified lysine crotonylation (Kcr) as a novel histone lysine acylation (Tan et al., 2011). Histone crotonylation represents an evolutionarily conserved histone mark which consists in the addition of a crotonyl group from crotonyl-coenzyme A to lysine residues, that similar to acetylation, modifies the charge of the histones (Tan et al., 2011; Fontecha-Barriuso et al., 2018). Nonetheless, the genomic pattern and function of histone crotonylation differs from histone acetylation (Tan et al., 2011). Histone crotonylation mainly marks active promoters and potential enhancers, but the impact on gene expression is unclear, as it can activate or repress gene transcription (Tan et al., 2011; Fellows et al., 2018).

### Histone Crotonyltranferases

**Figure 1:**
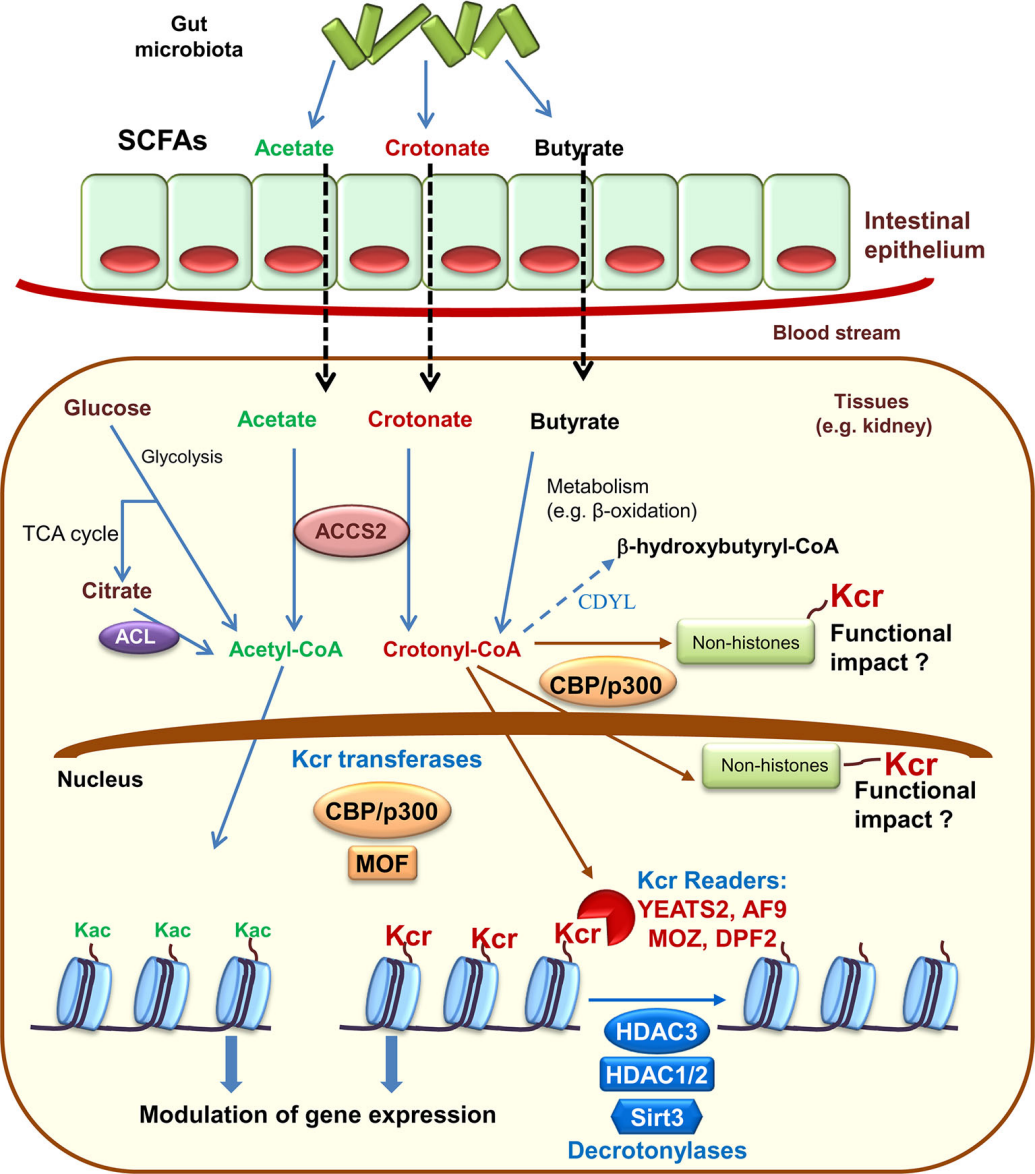
Histone crotonylation: enzymes and modulators. The gut microbiota is a source of short chain fatty acids (SCFAs) that inside cells may be metabolized to acetyl-CoA or crotonyl-CoA. These are the precursors that enzymes may use to promote lysine acetylation (Kac) or lysine crotonylation (Kcr) of histone and non-histone proteins. Crotonylated proteins have now been found within the nucleus and the cytoplasm. Already characterized crotonyltransferases (also termed crotonylases) include CBP/P300 and MOF, while histone decrotonylases include some histone deacetylases (HDAC) and sirtuin 3 (Sirt3). Kcr readers, proteins that identify Kcr in histones, include YEATS domain human proteins YEATS2 and AF9 as well as DPF family proteins MOZ and DPF2. Chromodomain Y-like (CDYL) negatively regulates histone crotonylation acting as a crotonyl-CoA hydratase that converts crotonyl-CoA required for Kcr into β-hydroxybutyryl-CoA. TCA, tricarboxylic acid; ACL, ATP citrate lyase; ACCS2, acyl-CoA Synthetase Short Chain Family Member 2.

Histone crotonylation shares enzyme regulators with histone acetylation. Specifically, the histone acetyltransferase CBP/P300 provides the bulk of histone crotonyltransferase (HCT) (also termed histone crotonylase) activity in mammalian cells (Liu X. et al., 2017). The evolutionary conserved MOF, from the MYST family of histone acetyltransferases, also has HCT activity, as well as its yeast homologue (Esa1) (Chen et al., 2015; Liu X. et al., 2017). Additionally, similar to other lysine acylations, histone crotonylation has been proposed to occur non-enzymatically (Wagner and Hirschey, 2014; Sabari et al., 2017). The enzymatic activity of CBP/P300 over crotonylation or acetylation depends on the levels of the precursors acetyl-CoA and crotonyl-CoA. Since intracellular acetyl-CoA is more abundant that crotonyl-CoA, the rate of crotonylation is more sensitive to changes of precursor levels (Sabari et al., 2015). In this line, while global histone acetylation is not affected by changes of sodium acetate availability, the levels of global histone crotonylation dramatically increase with increasing levels of sodium crotonate. This has physiological relevance, since crotonate addition also promotes gene expression *in vivo* and *in vitro* (Sabari et al., 2015; Ruiz-Andres et al., 2016a). Crotonate, the short-chain fatty acid (SCFA) precursor of crotonyl-CoA, is mainly produced by the colon microbiota (Stilling et al., 2016). Circulating SCFA (acetate, crotonate, butyrate, and propionate) are taken up by tissues and converted into acyl-CoA by the acyl-CoA Synthetase Short Chain Family Member 2 (ACSS2) or eventually yield crotonyl-CoA through different metabolic pathways such as fatty acid β-oxidation (Sabari et al., 2015; Rivière et al., 2016) (**Figure 1**). In this line, microbiota depletion decreases histone crotonylation in colon supporting the concept that microbiota may modulate epigenetic modifications (Fellows et al., 2018). Additionally, intracellular acetyl-CoA, generated during glycolysis, may also influence the extent of histone crotonylation. Under conditions of acetyl-CoA depletion, caused either by low glucose levels or by glucose carbons being directed away from ATP citrate lyase (ACL), other acyl-CoAs, such as crotonyl-CoA, will face less competition for acyl-transferase activity and this could also lead to epigenetic changes (Sabari et al., 2017).

### Kcr Readers

Histone covalent modifications are recognized by chromatin-binding protein modules, so-called “readers”. Acetyl lysine (Kac) residues are recognized mainly by bromodomains, YEATS domains, and double PHD-finger domains (DPF) (Liu X. et al., 2017). Bromodomains barely have affinity for long acyl modifications like Kcr sites (Andrews et al., 2016), although TAF1 can recognize them with low affinity (Flynn et al., 2015). By contrast, the evolutionarily conserved YEATS domain is a family of Kcr readers in yeast (Andrews et al., 2016). Indeed, YEATS domain human proteins YEATS2 and AF9 have higher affinity for Kcr sites than for shorter acyl-groups such as acetyl (Li et al., 2016; Zhao et al., 2016). Likewise, DPF family proteins MOZ and DPF2 recognize a wide range of histone lysine acylations with a strong preference for Kcr (Xiong et al., 2016).

### Histone Decrotonylases

HDACs also have decrotonylase activity. In mammals, there are 11 metal-dependent HDACs divided into class I (HDACs 1–3, 8), class II (HDAC 4–7, 9, 10), and class IV (HDAC 11) and seven sirtuins (Sirt1-7) (Lin et al., 2012; Lee, 2013). Class I HDACs are the main histone decrotonylases (Wei et al., 2017a). HDAC inhibitors such as suberoylanilide hydroxamic acid (SAHA, vorinostat) may enhance Kcr by inhibiting class I HDACs (Wu et al., 2017). The 3 µM vorinostat concentration used in these studies is within the range reached *in vivo* during the therapeutic use of the drug in humans (1.2 ± 0.62 µM) (https://www.accessdata.fda.gov/drugsatfda_docs/label/2011/021991s002lbl.pdf). HDAC1/2 containing complexes are critical regulators of histone Kcr (Kelly et al., 2018). Thus, genetic deletion of HDAC1/2 reduced total decrotonylase activity by 85%. Differences from prior studies could be related to the analysis of cells (Kelly et al., 2018) rather than of recombinant enzymes *in vitro* that described HDC3 decrotonylase activity (Tan et al., 2011; Madsen and Olsen, 2012), although there is the distinct possibility that different enzymes are more important in different cell types and environmental contexts. The histone decrotonylase activity of HDACs allows a further mechanism by which microbiota could increase crotonylation: generation of butyrate, an HDAC inhibitor (Shimazu et al., 2013). Although Sirt1 and Sirt3 have robust deacetylase activity (Houtkooper et al., 2012), they can also catalyze the hydrolysis of lysine crotonylated histones, but only Sirt3 regulates Kcr dynamics and gene transcription in living cells (Bao et al., 2014).

### Other Regulators of Kcr

There are additional pathways that may regulate Kcr. Chromodomain Y-like (CDYL) negatively regulates histone crotonylation acting as a crotonyl-CoA hydratase that converts crotonyl-CoA required for Kcr into β-hydroxybutyryl-CoA.

### Kcr vs Kac

Given the overlap between some Kcr and Kac sites in histones and shared modulators, initial concerns were raised as to the relevance of Kcr. It is now clear that Kcr and Kac are mechanistically and functionally different. Kcr is critically important for global transcription in mammalian cells (Wei et al., 2017b). Specifically, in both human somatic and mouse male germ cell genomes, histone Kcr marks either active promoters or potential enhancers (Tan et al., 2011). Kcr but not Kac preferentially marked “escapee genes” during post-meiotic sex inactivation in mouse testis (Montellier et al., 2012). Moreover, p300-catalyzed histone crotonylation directly stimulates transcription to a greater degree than histone acetylation (Sabari et al., 2015). Furthermore, CBP/p300 mutants may be deficient in HAT activity but competent for HCT activity (Liu X. et al., 2017). Indeed, YEATS domains and DPF proteins showed a greater affinity for Kcr over Kac and by two different molecular mechanisms (Li et al., 2016; Xiong et al., 2016). Finally, Kcr are more resistant to deacetylases than Kac, offering a better chance to resist repression and enabling a robust and productive transcription (Rousseaux and Khochbin, 2015).

## Histone Crotonylation in Non-Renal Tissues

Histone crotonylation was initially well characterized in human somatic and male germ cells (Tan et al., 2011). Recently, histone crotonylation has been observed in different cells and tissues but its function over gene expression has been only explored outside the kidney in gametogenesis, reprogramming of pluripotent stem-cells and colon epithelial cells.

### Gametogenesis and Pluripotent Stem Cells

Histone crotonylation, but not acetylation, is a specific mark of active promoters or potential enhancers of sex chromosomes-linked genes in male haploid cells immediately following meiosis and is associated with the release of transcriptional repressors in such sex chromosomes, which is considered an important element in the programming of the genome in the postmeiotic phase of spermatogenesis (Tan et al., 2011; Montellier et al., 2012). In this regard, CDYL decreased histone crotonylation and also sperm motility and fertility in mice (Liu S. et al., 2017). Histone crotonylation also favors the activation of pluripotency-inducing genes, maintenance of telomeres, and cell reprogramming during the chemical induction of pluripotent stem cells (CiPSCs) (Fu et al., 2018). In this line, in embryonic stem cells, Kcr and the expression of genes required for pluripotency were decreased by overexpression of mutant HDACs with impaired HDAC function but intact histone decrotonylase activity (Wei et al., 2017a).

### Gut Cells

In colon epithelial cells, crotonylated histones, more concretely H3K18cr, was associated to transcription start sites of genes involved in cancer, suggesting that deregulation of histones crotonylation may be linked to cancer. Butyrate from gut microbiota inhibited HDACs with decrotonylase activity, thus modifying the level of gut histone crotonylation and linking the gut microbiota with local tissue histone crotonylation (Fellows et al., 2018). In addition, microbiota depletion resulted in increased expression of HDAC2 (Fellows et al., 2018), an enzyme with decrotonylase activity and also related to tumorigenesis in colon cancer (Zhu et al., 2004) and with the progression from adenoma to carcinoma (Ashktorab et al., 2009). Several cancer-associated proteins are subject to Kcr by CBP/p300 (Huang et al., 2018). On the other hand, HDAC inhibitors induce the intestinal mucosa expression of ACSS2, which favors latent HIV reactivation through histone crotonylation (Jiang et al., 2018).

## Lysine Crotonylation Beyond Histones

Kcr has been identified in non-histone proteins. Studies using specific antibody enrichment followed by high resolution mass spectrometry and intensive bioinformatics analysis have demonstrated that Kcr occurs in a large number of proteins, predominantly in nuclei, but also in the cytoplasm (Wei et al., 2017b; Wu et al., 2017; Xu et al., 2017). Crotonylation was promoted by exposure to crotonate or CBP/p300 overexpression (Wei et al., 2017b). Crotonylated non-histone proteins have been characterized in different cell types such as Hela cells, H1299 cells (Wei et al., 2017b; Xu et al., 2017), HTC 116 (Huang et al., 2018), and in tissues such as lung, kidney, liver, colon, uterus, ovary, and brain of mice (Tweedie-Cullen et al., 2012; Xu et al., 2017).

Crotonylation of non-histone proteins is thought to be important in various biological processes, such as RNA splicing, gene expression, chromatin organization, nucleic acid metabolism, and the cell cycle (Wei et al., 2017b). However, the requirement of crotonylation for most of these putative cellular functions has not been formally tested. HDAC1 is among the crotonylated non-histone proteins in HELA cells treated with crotonate and HDAC1 crotonylation leads to a reduced deacetylase activity (Wei et al., 2017b).

## Histone Crotonylation in Kidney Injury

**Figure 2:**
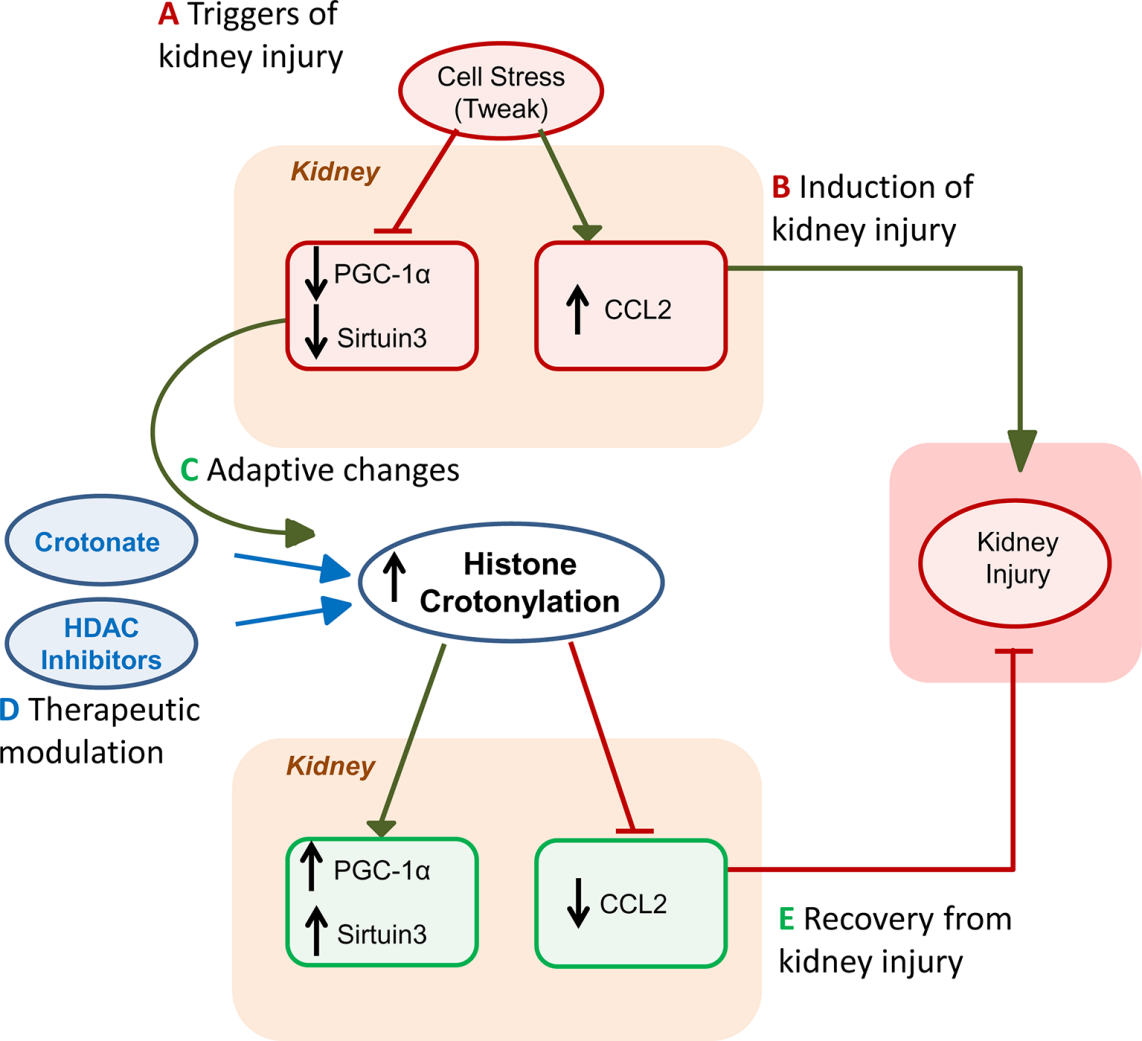
Role of histone crotonylation in acute kidney injury (AKI). The figures depict the different stages of kidney injury and the integration of spontaneous histone crotonylation or therapeutic drug-induced histone crotonylation. **(A)** Triggers of kidney injury, such as diverse forms of cell stress, including the proinflammatory cytokine TWEAK, cause lethal or sublethal (e.g. decreased expression of the master regulator of mitochondrial biogenesis PGC1α or sirtuin 3, Sirt3) kidney cell injury and elicit a proinflammatory response (e.g. chemokine synthesis) (Morigi et al., 2015; Martin-Sanchez et al., 2018; Fontecha-Barriuso et al., 2019). **(B)** This will induce kidney injury that may be magnified by recruitment of inflammatory cells (Ruiz-Andres et al., 2016b). **(C)** During AKI, global kidney histone crotonylation increases and this may be further increased by treatment with crotonate (Ruiz-Andres et al., 2016a). Decreased expression of decrotonylases, such as Sirt3 may contribute to increased global histone crotonylation. Based on crotonate therapy results, we hypothesize that the overall impact of increased global histone crotonylation supposes a brake in the kidney injury process, and, thus, a beneficial adaptive process. **(D)** As already indicated, treatment with crotonate improves kidney injury and restores PGC1α or Sirt3 expression, decreasing chemokine production. Based on new knowledge of the therapeutic effects of HDAC inhibitors on kidney injury *in vivo* and in culture (Chen et al., 2018; Fernandez-Fernandez et al., 2018), we now hypothesize that part of this beneficial effect of HDAC inhibitors may be related to their role in crotonylation regulation, rather than or in addition to inhibition of deacetylase activity. **(E)** Eventually, either spontaneously or following acceleration of recovery by therapy, kidney function recovers.

Kidney injury is probably the best characterized disease condition so far in terms of extent and function of histone crotonylation (Justo et al., 2006) (**Figure 2**). Histone Kcr was observed in tubular cells from healthy mouse and human kidney and nephrotoxic AKI was associated with a global increase in kidney histone crotonylation (Ruiz-Andres et al., 2016a). Likewise, in cultured proximal tubule epithelial cell cultures, stimulation with the proinflammatory cytokine TWEAK, a driver of kidney injury (Sanz et al., 2008; Ruiz-Andres et al., 2015), increased global histone Kcr (Ruiz-Andres et al., 2016a). Additionally, crotonate supplementation increased global histone Kcr in cultured tubular cells and in the kidneys *in vivo*. Thus, levels of histone crotonylation in kidney tubular cells may be modified by cell stress or by crotonate administration.

The increased histone crotonylation in cells stimulated with TWEAK and in AKI kidneys was associated with decreased PGC1α and Sirt-3 expression and with increased CCL2 expression, a chemokine which contributes to kidney inflammation (Ruiz-Andres et al., 2016a). PGC1α and Sirt-3 have nephroprotective actions (Kong et al., 2010; Morigi et al., 2015; Fontecha-Barriuso et al., 2019). Besides, Sirt3 and PGC1α regulate each other. PGC1α is a transcription factor and a major regulator of gluconeogenesis, as well as driver of mitochondrial biogenesis, and Sirt3 is a mediator of the effect of PGC1α on mitochondrial biogenesis (Van Beneden et al., 2011). The increased histone crotonylation induced by crotonate administration in mice and in cultured tubular kidney cells resulted in an increased PGC1α and sirtuin-3 expression, as well as a decrease of CCL2 expression. That is, the systemic administration of crotonate prevents the decrease of PGC1α and sirtuin-3 expression, and the increase of CCL2 expression in mice with AKI and this was associated to protection from experimental nephrotoxic AKI and preservation of renal function (Justo et al., 2006). Taken together, the cell culture evidence that crotonate increased histone crotonylation and prevented proinflammatory responses while preserving nephroprotective gene expression, the fact that it had similar actions *in vivo* in AKI and that it protected from AKI, suggests that crotonate may have a potential therapeutic effect on kidney damage, and more specifically in AKI, by increasing histone crotonylation. If this hypothesis is correct, then the spontaneous global increase in histone crotonylation during AKI may be considered part of an adaptive mechanism, even allowing for potential discrepant level of crotonylation in specific genes. In this regard, the previously described beneficial effects of HDAC inhibitors such as valproic acid or trichostatin, in kidney damage (Moreno et al., 2011; Van Beneden et al., 2013), should be viewed in the context of their potential to induce histone crotonylation (Madsen and Olsen, 2012; Wei et al., 2017a; Kelly et al., 2018) and not just through the light of their impact on histone lysine acetylation. Some examples of beneficial effects of HDAC inhibitors that have been ascribed to modulation of histone acetylation but may in fact be related to increase histone crotonylation include preservation of the expression of the antiaging and nephroprotective gene Klotho despite the presence of Klotho suppressors such as inflammatory cytokines and albuminuria, and similar results were observed with PGC1α expression (Chen et al., 2018; Fernandez-Fernandez et al., 2018; Fontecha-Barriuso et al., 2019). This hypothesis should be addressed through specifically designed experiments.

In peripheral blood mononuclear cells from dialysis patients or healthy subjects, liquid chromatography–mass spectrometry identified 347 crotonylated proteins (Lin et al., 2020). A total of 345 proteins were differentially expressed between hemodialysis patients and controls, 93 of them upregulated and 252 downregulated. However, functional enrichment analysis did not show significant differences between upregulated and downregulated proteins and no direct relation was observed between Kcr ratio and levels of expression.

## Summary and Future Perspectives

In summary, histone lysine crotonylation is a recently described posttranslational histone modification which, like previously described ones, has the potential to regulated gene expression. To date, its function in disease has been more extensively investigated in kidney injury. AKI and tubular cell stress in culture are characterized by increased global histone crotonylation. While it is conceivable that this is the end-result of differential crotonylation of many different genes, with very different impact on the expression of these genes, supplementing crotonate both increased kidney histone crotonylation and was nephroprotective and this was associated with potential beneficial changes in the expression of nephroprotective and proinflammatory genes. This opens the door to explore therapeutic strategies based on the modulation of histone crotonylation that, besides supplementing crotonate, may also include modulation of the microbiota and the use of HDAC inhibitors. In this regard the literature on the nephroprotective effects of HDAC inhibition should be re-examined from the point of view of their potential regulation of histone crotonylation. A detailed exploration of the role of histone crotonylation in kidney disease may facilitate the study of its impact in health and disease of other organs. Finally, the regulation and role of non-histone crotonylation remains essentially unexplored. A recent manuscript using a proteomics approach identified 155 upregulated and 198 downregulated crotonylated proteins in peripheral blood mononuclear cells from IgA nephropathy patients, of which only three were histones. Since only six patients were studied, whether these changes are linked to IgA nephropathy itself, to CKD or to other factors, remains unclear (Huang et al., 2012). In any case, this manuscript identifies the potential of a systems biology approach and eventual bioinformatics and machine learning analyses in advancing the field. Thus, a systems biology analysis of the impact of HDAC inhibitors in AKI may identify new biomarkers or therapeutic targets.

## Author Contributions

JM-M, MF-B, DM-S, MS-N and MR-O assessed the articles and their relevance to the above topics and wrote the manuscript. AO and AS supervised and drafted the manuscript and are the corresponding authors.

## Funding

Sources of support: FIS/FEDER funds (PI15/00298, CP14/00133, PI16/02057, PI16/01900, PI18/01386, PI19/00588, PI19/00815, DTS18/00032, ERA-PerMed-JTC2018 (KIDNEY ATTACK AC18/00064 and PERSTIGAN AC18/00071), ISCIII-RETIC REDinREN RD016/0009), Sociedad Española de Nefrología, FRIAT, Comunidad de Madrid en Biomedicina B2017/BMD-3686 CIFRA2-CM. Salary support: ISCIII Miguel Servet and to AS and MS-N, ISCIII Sara Borrell to JM-M and Comunidad de Madrid (B2017/BMD-3686 CIFRA2-CM) to MF-B and DM-S. The authors declare no conflict of interest.

## Conflict of Interest

AS, AO, and M-SN have a patent on the use of crotonate to prevent or treat acute kidney injury.

The remaining authors declare that the research was conducted in the absence of any commercial or financial relationships that could be construed as a potential conflict of interest.

## References

[B49] Andrews F. H. Shinsky S. A. Shanle E. K. Bridgers J. B. Gest A. Tsun I. K. (2016). The Taf14 YEATS domain is a reader of histone crotonylation. Nat. Chem. Biol. 12, 396–398. 10.1038/nchembio.2065 27089029 PMC4871749

[B69] Ashktorab H. Belgrave K. Hosseinkhah F. Brim H. Nouraie M. Takkikto M. (2009). Global histone H4 acetylation and HDAC2 expression in colon adenoma and carcinoma. Dig. Dis. Sci. 54, 2109–2117. 10.1007/s10620-008-0601-7 19057998 PMC2737733

[B62] Bao X. Wang Y. Li X. Xiao-Meng L. Liu Z. Yang T. (2014). Identification of ‘erasers’ for lysine crotonylated histone marks using a chemical proteomics approach. eLife 3, e02999. 10.7554/eLife.02999 PMC4358366 25369635

[B23] Bechtel W. McGoohan S. Zeisberg E. M. Müller G. A. Kalbacher H. Salant D. J. (2010). Methylation determines fibroblast activation and fibrogenesis in the kidney. Nat. Med. 16, 544–550. 10.1038/nm.2135 20418885 PMC3106179

[B11] Beckerman P. Ko Y. A. Susztak K. (2014). Epigenetics: a new way to look at kidney diseases. Nephrol. Dial Transplant. 29, 1821–1827. 10.1093/ndt/gfu026 24675284 PMC4173816

[B14] Campos E. I. Reinberg D. (2009). Histones: annotating chromatin. Annu. Rev. Genet. 43, 559–599. 10.1146/annurev.genet.032608.103928 19886812

[B5] Chawla L. S. Eggers P. W. Star R. A. Kimmel P. L. (2014). Acute kidney injury and chronic kidney disease as interconnected syndromes. N Engl. J. Med. 371, 58–66. 10.1056/NEJMra1214243 24988558 PMC9720902

[B43] Chen Q. Y. Costa M. Sun H. (2015). Structure and function of histone acetyltransferase MOF. AIMS Biophys. 2, 555–569. 10.3934/biophy.2015.4.555 28503659 PMC5425159

[B84] Chen W. Tang D. Xu Y. Zou Y. Sui W. Dai Y. (2018). Comprehensive analysis of lysine crotonylation in proteome of maintenance hemodialysis patients. Med. (Baltimore) 97, e12035. 10.1097/MD.0000000000012035 PMC6156053 30212933

[B12] Davey C. A. Sargent D. F. Luger K. Maeder A. W. Richmond T. J. (2002). Solvent mediated interactions in the structure of the nucleosome core particle at 1.9 a resolution. J. Mol. Biol. 319, 1097–1113. 10.1016/S0022-2836(02)00386-8 12079350

[B41] Fellows R. Denizot J. Stellato C. Cuomo A. Jain P. Stoyanova E. (2018). Microbiota derived short chain fatty acids promote histone crotonylation in the colon through histone deacetylases. Nat. Commun. 9, 105. 10.1038/s41467-017-02651-5 29317660 PMC5760624

[B83] Fernandez-Fernandez B. Izquierdo M. C. Valino-Rivas L. Nastou D. Sanz A. B. Ortiz A. (2018). Albumin downregulates Klotho in tubular cells. Nephrol. Dialysis Transplant. 33, 1712–1722. 10.1093/ndt/gfx376 29425318

[B50] Flynn E. M. Huang O. W. Poy F. Oppikofer M. Bellon S. F. Tang Y. (2015). A Subset of Human Bromodomains Recognizes Butyryllysine and Crotonyllysine Histone Peptide Modifications. Structure 23, 1801–1814. 10.1016/j.str.2015.08.004 26365797

[B26] Fontecha-Barriuso M. Martin-Sanchez D. Ruiz-Andres O. Poveda J. Sanchez-Niño M. D. Valiño-Rivas L. (2018). Targeting epigenetic DNA and histone modifications to treat kidney disease. Nephrol. Dialysis Transplant. 33, 1875–1886. 10.1093/ndt/gfy009 29534238

[B77] Fontecha-Barriuso M. Martín-Sánchez D. Martinez-Moreno J. M. Carrasco S. Ruiz-Andrés O. Monsalve M. (2019). PGC-1α deficiency causes spontaneous kidney inflammation and increases the severity of nephrotoxic AKI. J. Pathol. 1, 65–78. 10.1002/path.5282 30982966

[B67] Fu H. Tian C. L. Ye X. Sheng X. Wang H. Liu Y. (2018). Dynamics of Telomere Rejuvenation during Chemical Induction to Pluripotent Stem Cells. Stem Cell Rep. 11, 70–87. 10.1016/j.stemcr.2018.05.003 PMC6066961 29861168

[B22] Guo C. Pei L. Xiao X. Wei Q. Chen J. K. Ding H. F. (2017). DNA methylation protects against cisplatin-induced kidney injury by regulating specific genes, including interferon regulatory factor 8. Kidney Int. 92, 1194–1205. 10.1016/j.kint.2017.03.038 28709638 PMC5651199

[B33] Hewitson T. D. Holt S. G. Tan S. J. Wigg B. Samuel C. S. Smith E. R. (2017). Epigenetic Modifications to H3K9 in Renal Tubulointerstitial Cells after Unilateral Ureteric Obstruction and TGF-β1 Stimulation. Front. Pharmacol. 8, 307. 10.3389/fphar.2017.00307 28611663 PMC5447091

[B61] Houtkooper R. H. Pirinen E. Auwerx J. (2012). Sirtuins as regulators of metabolism and healthspan. Nat. Rev. Mol. Cell Biol. 13, 225–238. 10.1038/nrm3293 22395773 PMC4872805

[B86] Huang N. Tan L. Xue Z. Cang J. Wang H. (2012). Reduction of DNA hydroxymethylation in the mouse kidney insulted by ischemia reperfusion. Biochem. Biophys. Res. Commun. 422, 697–702. 10.1016/j.bbrc.2012.05.061 22627137

[B32] Huang J. Wan D. Li J. Chen H. Huang K. Zheng L. (2015). Histone acetyltransferase PCAF regulates inflammatory molecules in the development of renal injury. Epigenetics 10, 62–72. 10.4161/15592294.2014.990780 25496441 PMC4622516

[B70] Huang H. Wang D. L. Zhao Y. (2018). Quantitative Crotonylome Analysis Expands the Roles of p300 in the Regulation of Lysine Crotonylation Pathway. Proteomics 18, e1700230. 10.1002/pmic.201700230 29932303 PMC6420807

[B71] Jiang G. Nguyen D. Archin N. M. Yukl S. A. Méndez-Lagares G. Tang Y. (2018). HIV latency is reversed by ACSS2-driven histone crotonylation. J. Clin. Invest. 128, 1190–1198. 10.1172/JCI98071 29457784 PMC5824862

[B74] Justo P. Sanz A. B. Sanchez-Nino M. D. Sanchez-Nino M. D. Winkles J. A. Lorz C. Egido J. (2006). Cytokine cooperation in renal tubular cell injury: The role of TWEAK. Kidney Int. 70, 1750–1758. 10.1038/sj.ki.5001866 17003819

[B58] Kelly R. D. W. Chandru A. Watson P. J. Song Y. Blades M. Robertson N. S. (2018). Histone deacetylase (HDAC) 1 and 2 complexes regulate both histone acetylation and crotonylation in vivo. Sci. Rep. 8, 14690. 10.1038/s41598-018-32927-9 30279482 PMC6168483

[B7] Kida Y. Ieronimakis N. Schrimpf C. Reyes M. Duffield J. S. (2013). EphrinB2 reverse signaling protects against capillary rarefaction and fibrosis after kidney injury. J. Am. Soc. Nephrol. 24, 559–572. 10.1681/ASN.2012080871 23492730 PMC3609137

[B1] Kidney Disease (2012). Improving Global Outcomes (KDIGO) AKI Work Group. KDIGO Clinical Practice Guideline for Acute Kidney Injury. Kidney Int. Suppl. 2, 1–138. 10.1038/kisup.2012.6

[B2] Kidney Disease (2013). Improving Global Outcomes (KDIGO) CKD Work Group. KDIGO 2012 Clinical Practice Guideline for the Evaluation and Management of Chronic Kidney Disease. Kidney Int. Suppl. 3, 1–150. 10.1038/kisup.2012.73

[B87] Ko Y. A. Mohtat D. Suzuki M. Park A. S. Izquierdo M. C. Han S. Y. (2013). Cytosine methylation changes in enhancer regions of core pro-fibrotic genes characterize kidney fibrosis development. Genome Biol. 14, R108. 10.1186/gb-2013-14-10-r108 24098934 PMC4053753

[B79] Kong X. Wang R. Xue Y. Liu X. Zhang H. Chen Y. (2010). Sirtuin 3, a new target of PGC-1alpha, plays an important role in the suppression of ROS and mitochondrial biogenesis. PloS One 5, e11707. 10.1371/journal.pone.0011707 20661474 PMC2908542

[B54] Lee S. (2013). Post-translational modification of proteins in toxicological research: focus on lysine acylation. Toxicol. Res. 29, 81–86. 10.5487/TR.2013.29.2.081 24278632 PMC3834447

[B38] Li H. F. Cheng C. F. Liao W. J. Lin H. Yang R. B. (2010). ATF3-mediated epigenetic regulation protects against acute kidney injury. J. Am. Soc. Nephrol. 21, 1003–1013. 10.1681/ASN.2009070690 20360311 PMC2900964

[B52] Li Y. Sabari B. R. Panchenko T. Wen H. Zhao D. Guan H. (2016). Molecular Coupling of Histone Crotonylation and Active Transcription by AF9 YEATS Domain. Mol. Cell 62, 181–193. 10.1016/j.molcel.2016.03.028 27105114 PMC4841940

[B28] Li L. X. Fan L. X. Zhou J. X. Grantham J. J. Calvet J. P. Sage J. (2017). Lysine methyltransferase SMYD2 promotes cyst growth in autosomal dominant polycystic kidney disease. J. Clin. Invest. 127, 2751–2764. 10.1172/JCI90921 28604386 PMC5490754

[B55] Lin H. Su X. He B. (2012). Protein lysine acylation and cysteine succination by intermediates of energy metabolism. ACS Chem. Biol. 7, 947–960. 10.1021/cb3001793 22571489 PMC3376250

[B85] Lin H. Tang D. Xu Y. Zhang R. Ou M. Zheng F. (2020). Quantitative analysis of protein crotonylation identifies its association with immunoglobulin A nephropathy. Mol. Med. Rep. 21, 1242–1250. 10.3892/mmr.2020.10931 32016442 PMC7002971

[B42] Liu X. Wei W. Liu Y. Yang X. Wu J. Zhang Y. (2017). MOF as an evolutionarily conserved histone crotonyltransferase and transcriptional activation by histone acetyltransferase-deficient and crotonyltransferase-competent CBP/p300. Cell Discovery 3, 17016. 10.1038/celldisc.2017.16 28580166 PMC5441097

[B66] Liu S. Yu H. Liu Y. Liu X. Zhang Y. Bu C. (2017). Chromodomain Protein CDYL Acts as a Crotonyl-CoA Hydratase to Regulate Histone Crotonylation and Spermatogenesis. Mol. Cell 67, 853–866.e855. 10.1016/j.molcel.2017.07.011 28803779

[B13] Luger K. Mäder A. W. Richmond R. K. Sargent D. F. Richmond T. J. (1997). Crystal structure of the nucleosome core particle at 2.8 A resolution. Nature 389, 251–260. 10.1038/38444 9305837

[B59] Madsen A. S. Olsen C. A. (2012). Profiling of substrates for zinc-dependent lysine deacylase enzymes: HDAC3 exhibits decrotonylase activity in vitro. Angew Chem. Int. Ed Engl. 51, 9083–9087. 10.1002/anie.201203754 22890609

[B91] Martin-Sanchez D. Fontecha-Barriuso M. Carrasco S. Sanchez-Niño M. D. Mässenhausen A. V. Linkermann A. (2018). TWEAK and RIPK1 mediate a second wave of cell death during AKI. Proc. Natl. Acad. Sci. U. S. A 115, 4182–4187. 10.1073/pnas.1716578115 29588419 PMC5910825

[B35] Marumo T. Hishikawa K. Yoshikawa M. Fujita T. (2008). Epigenetic regulation of BMP7 in the regenerative response to ischemia. J. Am. Soc. Nephrol. 19, 1311–1320. 10.1681/ASN.2007091040 18322163 PMC2440290

[B10] Mohtat D. Susztak K. (2010). Fine tuning gene expression: the epigenome. Semin. Nephrol. 30, 468–476. 10.1016/j.semnephrol.2010.07.004 21044758 PMC3164355

[B64] Montellier E. Rousseaux S. Zhao Y. Khochbin S. (2012). Histone crotonylation specifically marks the haploid male germ cell gene expression program: post-meiotic male-specific gene expression. Bioessays 34, 187–193. 10.1002/bies.201100141 22170506

[B82] Moreno J. A. Izquierdo M. C. Sanchez-Niño M. D. Suárez-Alvarez B. Lopez-Larrea C. Jakubowski A. (2011). The inflammatory cytokines TWEAK and TNFα reduce renal klotho expression through NFκB. J. Am. Soc. Nephrol. 22, 1315–1325. 10.1681/ASN.2010101073 21719790 PMC3137579

[B16] Morera L. Lübbert M. Jung M. (2016). Targeting histone methyltransferases and demethylases in clinical trials for cancer therapy. Clin. Epigenet. 8, 57. 10.1186/s13148-016-0223-4 PMC4877953 27222667

[B78] Morigi M. Perico L. Rota C. Longaretti L. Conti S. Rottoli D. (2015). Sirtuin 3-dependent mitochondrial dynamic improvements protect against acute kidney injury. J. Clin. Invest. 125, 715–726. 10.1172/JCI77632 25607838 PMC4319434

[B31] Munshi R. Johnson A. Siew E. D. Ikizler T. A. Ware L. B. Wurfel M. M. (2011). MCP-1 gene activation marks acute kidney injury. J. Am. Soc. Nephrol. 22, 165–175. 10.1681/ASN.2010060641 21071523 PMC3014045

[B29] Naito M. Bomsztyk K. Zager R. A. (2008). Endotoxin mediates recruitment of RNA polymerase II to target genes in acute renal failure. J. Am. Soc. Nephrol. 19, 1321–1330. 10.1681/ASN.2007121368 18417719 PMC2440304

[B37] Naito M. Bomsztyk K. Zager R. A. (2009). Renal ischemia-induced cholesterol loading: transcription factor recruitment and chromatin remodeling along the HMG CoA reductase gene. Am. J. Pathol. 174, 54–62. 10.2353/ajpath.2009.080602 19095962 PMC2631318

[B17] Nguyen A. T. Zhang Y. (2011). The diverse functions of Dot1 and H3K79 methylation. Genes Dev. 25, 1345–1358. 10.1101/gad.2057811 21724828 PMC3134078

[B90] Ortiz A. Husi H. Gonzalez-Lafuente L. Valiño-Rivas L. Fresno M. Sanz A. B. (2016). Mitogen-Activated Protein Kinase 14 Promotes AKI. J. Am. Soc. Nephrol. 28, 823–836. 10.1681/ASN.2015080898 27620989 PMC5328147

[B4] Ortiz A. Sanchez-Niño M. D. Crespo-Barrio M. De-Sequera-Ortiz P. Fernández-Giráldez E. García-Maset R. (2019). The Spanish Society of Nephrology (SENEFRO) commentary to the Spain GBD 2016 report: Keeping chronic kidney disease out of sight of health authorities will only magnify the problem. Nefrologia 39, 29–34. 10.1016/j.nefro.2018.09.002 30503082

[B9] Perez-Gomez M. V. Sanchez-Niño M. D. Sanz A. B. Martín-Cleary C. Ruiz-Ortega M. Egido J. (2015). Horizon 2020 in Diabetic Kidney Disease: The Clinical Trial Pipeline for Add-On Therapies on Top of Renin Angiotensin System Blockade. J. Clin. Med. 4, 1325–1347. 10.3390/jcm4061325 26239562 PMC4485003

[B3] Perez-Gomez M. V. Bartsch L. A. Castillo-Rodriguez E. Fernandez-Prado R. Fernandez-Fernandez B. Martin-Cleary C. (2019). Clarifying the concept of chronic kidney disease for non-nephrologists. Clin. Kidney J. 12, 258–261. 10.1093/ckj/sfz007 30976406 PMC6452188

[B6] Polichnowski A. J. (2018). Microvascular rarefaction and hypertension in the impaired recovery and progression of kidney disease following AKI in preexisting CKD states. Am. J. Physiol. Renal Physiol. 315, F1513–F1518. 10.1152/ajprenal.00419.2018 30256130 PMC6336987

[B18] Ramakrishnan S. Pili R. (2013). Histone deacetylase inhibitors and epigenetic modifications as a novel strategy in renal cell carcinoma. Cancer J. 19, 333–340. 10.1097/PPO.0b013e3182a09e07 23867515 PMC3766322

[B48] Rivière A. Selak M. Lantin D. (2016). Bifidobacteria and Butyrate-Producing Colon Bacteria: Importance and Strategies for Their Stimulation in the Human Gut. Front. Microbiol. 7, 979. 10.3389/fmicb.2016.00979 27446020 PMC4923077

[B65] Rousseaux S. Khochbin S. (2015). Histone Acylation beyond Acetylation: Terra Incognita in Chromatin Biology. Cell J. 17, 1–6. 10.22074/cellj.2015.506 25870829 PMC4393657

[B76] Ruiz-Andres O. Suarez-Alvarez B. Sánchez-Ramos C. Monsalve M. Sanchez-Niño M. D. Ruiz-Ortega M. (2015). The inflammatory cytokine TWEAK decreases PGC-1α expression and mitochondrial function in acute kidney injury. Kidney Int. 89, 399–410. 10.1038/ki.2015.332 26535995

[B20] Ruiz-Andres O. Sanchez-Niño M. D. Cannata-Ortiz P. Ruiz-Ortega M. Egido J. Ortiz A. (2016a). Histone lysine crotonylation during acute kidney injury in mice. Dis. Model Mech. 9, 633–645. 10.1242/dmm.024455 27125278 PMC4920150

[B39] Ruiz-Andres O. Sanchez-Niño M. D. Moreno J. A. Ruiz-Ortega M. Ramos A. M. Sanz A. B. (2016b). Downregulation of kidney protective factors by inflammation: role of transcription factors and epigenetic mechanisms. Am. J. Physiol. Renal Physiol. 311, F1329–F1340. 10.1152/ajprenal.00487.2016 27760772

[B46] Sabari B. R. Tang Z. Huang H. Yong-Gonzalez V. Molina H. Kong H. E. (2015). Intracellular crotonyl-CoA stimulates transcription through p300-catalyzed histone crotonylation. Mol. Cell 58, 203–215. 10.1016/j.molcel.2015.02.029 25818647 PMC4501262

[B44] Sabari B. R. Zhang D. Allis C. D. Zhao Y. (2017). Metabolic regulation of gene expression through histone acylations. Nat. Rev. Mol. Cell Biol. 18, 90–101. 10.1038/nrm.2016.140 27924077 PMC5320945

[B75] Sanz A. B. Justo P. Sanchez-Nino M. D. Blanco-Colio L. M. Winkles J. A. Kreztler M. (2008). The cytokine TWEAK modulates renal tubulointerstitial inflammation. J. Am. Soc. Nephrol. 19, 695–703. 10.1681/ASN.2007050577 18235096 PMC2390965

[B34] Sayyed S. G. Gaikwad A. B. Lichtnekert J. Kulkarni O. Eulberg D. Klussmann S. (2010). Progressive glomerulosclerosis in type 2 diabetes is associated with renal histone H3K9 and H3K23 acetylation, H3K4 dimethylation and phosphorylation at serine 10. Nephrol. Dial Transplant. 25, 1811–1817. 10.1093/ndt/gfp730 20067909

[B60] Shimazu T. Hirschey M. D. Newman J. He W. Shirakawa K. Moan N. L. (2013). Suppression of oxidative stress by β-hydroxybutyrate, an endogenous histone deacetylase inhibitor. Science 339, 211–214. 10.1126/science.1227166 23223453 PMC3735349

[B88] Stenvinkel P. Karimi M. Johansson S. Axelsson J. Suliman M. Lindholm B. (2007). Impact of inflammation on epigenetic DNA methylation - a novel risk factor for cardiovascular disease? J. Int. Med. 261, 488–499. 10.1111/j.1365-2796.2007.01777.x 17444888

[B47] Stilling R. M. van de Wouw M. Clarke G. Stanton C. Dinan T. G. Cryan J. F. (2016). The neuropharmacology of butyrate: The bread and butter of the microbiota-gut-brain axis? Neurochem. Int. 99, 110–132. 10.1016/j.neuint.2016.06.011 27346602

[B40] Suarez-Alvarez B. Morgado-Pascual J. L. Rayego-Mateos S. Rodriguez M. Rodrigues-Diez R. Cannata-Ortiz P. (2017). Inhibition of Bromodomain and Extraterminal Domain Family Proteins Ameliorates Experimental Renal Damage. J. Am. Soc. Nephrol. 28, 504–519. 10.1681/ASN.2015080910 27436852 PMC5280004

[B24] Sun C. Y. Chang S. C. Wu M. S. (2012). Suppression of Klotho expression by protein-bound uremic toxins is associated with increased DNA methyltransferase expression and DNA hypermethylation. Kidney Int. 81, 640–650. 10.1038/ki.2011.445 22237753 PMC3306006

[B15] Susztak K. (2014). Understanding the epigenetic syntax for the genetic alphabet in the kidney. J. Am. Soc. Nephrol. 25, 10–17. 10.1681/ASN.2013050461 24179169 PMC3871782

[B25] Tampe B. Steinle U. Tampe D. Carstens J. L. Korsten P. Zeisberg E. M. (2017). Low-dose hydralazine prevents fibrosis in a murine model of acute kidney injury-to-chronic kidney disease progression. Kidney Int. 91, 157–176. 10.1016/j.kint.2016.07.042 27692563

[B19] Tan M. Luo H. Lee S. Jin F. Yang J. S. Montellier E. (2011). Identification of 67 histone marks and histone lysine crotonylation as a new type of histone modification. Cell 146, 1016–1028. 10.1016/j.cell.2011.08.008 21925322 PMC3176443

[B73] Tweedie-Cullen R. Y. Brunner A. M. Grossmann J. Mohanna S. Sichau D. Nanni P. (2012). Identification of combinatorial patterns of post-translational modifications on individual histones in the mouse brain. PloS One 7, e36980. 10.1371/journal.pone.0036980 22693562 PMC3365036

[B80] Van Beneden K. Geers C. Pauwels M. Mannaerts I. Verbeelen D. van Grunsven L. A. (2011). Valproic acid attenuates proteinuria and kidney injury. J. Am. Soc. Nephrol. 22, 1863–1875. 10.1681/ASN.2010111196 21868496 PMC3279948

[B81] Van Beneden K. Geers C. Pauwels M. Mannaerts I. Wissing K. M. Van den Branden C. (2013). Comparison of trichostatin A and valproic acid treatment regimens in a mouse model of kidney fibrosis. Toxicol. Appl. Pharmacol. 271, 276–284. 10.1016/j.taap.2013.05.013 23707763

[B45] Wagner G. R. Hirschey M. D. (2014). Nonenzymatic protein acylation as a carbon stress regulated by sirtuin deacylases. Mol. Cell 54, 5–16. 10.1016/j.molcel.2014.03.027 24725594 PMC4040445

[B56] Wei W. Liu X. Chen J. Gao S. Lu L. Zhang H. (2017a). Class I histone deacetylases are major histone decrotonylases: evidence for critical and broad function of histone crotonylation in transcription. Cell Res. 27, 898–915. 10.1038/cr.2017.68 28497810 PMC5518989

[B63] Wei W. Mao A. Tang B. Zeng Q. Gao S. Liu X. (2017b). Large-Scale Identification of Protein Crotonylation Reveals Its Role in Multiple Cellular Functions. J. Proteome Res. 16, 1743–1752. 10.1021/acs.jproteome.7b00012 28234478

[B57] Wu Q. Li W. Wang C. Fan P. Cao L. Wu Z. (2017). Ultradeep Lysine Crotonylome Reveals the Crotonylation Enhancement on Both Histones and Nonhistone Proteins by SAHA Treatment. J. Proteome Res. 16, 3664–3671. 10.1021/acs.jproteome.7b00380 28882038

[B53] Xiong X. Panchenko T. Yang S. Zhao S. Yan P. Zhang W. (2016). Selective recognition of histone crotonylation by double PHD fingers of MOZ and DPF2. Nat. Chem. Biol. 12, 1111–1118. 10.1038/nchembio.2218 27775714 PMC5253430

[B72] Xu W. Wan J. Zhan J. Li X. He H. Shi Z. (2017). Global profiling of crotonylation on non-histone proteins. Cell Res. 27, 946–949. 10.1038/cr.2017.60 28429772 PMC5518986

[B8] Yang B. Lan S. Dieudé M. Sabo-Vatasescu J. P. Karakeussian-Rimbaud A. Turgeon J. (2018). Caspase-3 Is a Pivotal Regulator of Microvascular Rarefaction and Renal Fibrosis after Ischemia-Reperfusion Injury. J. Am. Soc. Nephrol. 29, 1900–1916. 10.1681/ASN.2017050581 29925521 PMC6050936

[B30] Zager R. A. Johnson A. C. (2009). Renal ischemia-reperfusion injury upregulates histone-modifying enzyme systems and alters histone expression at proinflammatory/profibrotic genes. Am. J. Physiol. Renal Physiol. 296, F1032–F1041. 10.1152/ajprenal.00061.2009 19261745 PMC2681356

[B36] Zager R. A. Johnson A. C. Becker K. (2011). Acute unilateral ischemic renal injury induces progressive renal inflammation, lipid accumulation, histone modification, and “end-stage” kidney disease. Am. J. Physiol. Renal Physiol. 301, F1334–F1345. 10.1152/ajprenal.00431.2011 21921025 PMC3233867

[B89] Zhang Q. Liu L. Lin W. Yin S. Duan A. Liu Z. (2017). Rhein reverses Klotho repression via promoter demethylation and protects against kidney and bone injuries in mice with chronic kidney disease. Kidney Int. 91, 144–156. 10.1016/j.kint.2016.07.040 27692562

[B51] Zhao D. Guan H. Zhao S. Mi W. Wen H. Li Y. (2016). YEATS2 is a selective histone crotonylation reader. Cell Res. 26, 629–632. 10.1038/cr.2016.49 27103431 PMC4856769

[B21] Zhao Y. Ding C. Xue W. Ding X. Zheng J. Gao Y. (2017). Genome-wide DNA methylation analysis in renal ischemia reperfusion injury. Gene 610, 32–43. 10.1016/j.gene.2017.02.005 28189760

[B27] Zhou X. Zang X. Ponnusamy M. Masucci M. V. Tolbert E. Gong R. (2016). Enhancer of Zeste Homolog 2 Inhibition Attenuates Renal Fibrosis by Maintaining Smad7 and Phosphatase and Tensin Homolog Expression. J. Am. Soc. Nephrol. 27, 2092–2108. 10.1681/ASN.2015040457 26701983 PMC4926973

[B68] Zhu P. Martin E. Mengwasser J. Schlag P. Janssen K. P. Göttlicher M. (2004). Induction of HDAC2 expression upon loss of APC in colorectal tumorigenesis. Cancer Cell 5, 455–463. 10.1016/S1535-6108(04)00114-X 15144953

